# Effects of Long-Lasting High-Definition Transcranial Direct Current Stimulation in Chronic Disorders of Consciousness: A Pilot Study

**DOI:** 10.3389/fnins.2019.00412

**Published:** 2019-04-30

**Authors:** Yongkun Guo, Yang Bai, Xiaoyu Xia, Jinju Li, Xiaoli Wang, Yiwu Dai, Yuanyuan Dang, Jianghong He, Chunying Liu, Hui Zhang

**Affiliations:** ^1^Department of Neurosurgery, Zhengzhou Central Hospital Affiliated to Zhengzhou University, Zhengzhou, China; ^2^International Vegetative State and Consciousness Science Institute, Hangzhou Normal University, Hangzhou, China; ^3^Department of Basic Medical Science, School of Medicine, Hangzhou Normal University, Hangzhou, China; ^4^Department of Neurosurgery, PLA Army General Hospital, Beijing, China

**Keywords:** disorders of consciousness, high-definition transcranial direct current stimulation, electroencephalography, Coma Recovery Scale–Revised scores, coherence

## Abstract

Transcranial direct current stimulation (tDCS) recently was shown to benefit rehabilitation of patients with disorders of consciousness (DOC). However, high-Definition tDCS (HD-tDCS) has not been applied in DOC. In this study, we tried to use HD-tDCS protocol (2 mA, 20 min, the precuneus, and sustaining 14 days) to rehabilitate 11 patients with DOC. Electroencephalography (EEG) and Coma Recovery Scale–Revised (CRS-R) scores were recorded at before (T0), after a single session (T1), after 7 days’ (T2), and 14 days’ HD-tDCS (T3) to assess the modulation effects. EEG coherence was measured to evaluate functional connectivity during the experiment. It showed that 9 patients’ scores increased compared with the baseline. The central-parietal coherence significantly decreased in the delta band in patients with DOC. EEG coherence might be useful for assessing the effect of HD-tDCS in patients with DOC. Long-lasting HD-tDCS over the precuneus is promising for the treatment of patients with DOC.

## Introduction

Chronic disorders of consciousness (DOC) consist of vegetative state/ unresponsive wakefulness syndrome (VS/ UWS) and minimally conscious state (MCS) (Giacino et al., 2014). MCS is characterized by minimal but definite behavioral evidence of self or environmental awareness (Giacino et al., 2014). Recently, MCS was subcategorized into MCS- describing low-level behavioral responses and MCS+ describing high level behavioral responses (Bruno et al., 2012). Even though many pharmacological (i.e., amantadine, zolpidem) and non-pharmacological interventions (i.e., deep brain stimulation, spinal cord Stimulation, transcranial magnetic stimulation, median nerve electrical stimulation) have been assessed in the last decade, there remain few effective therapies for patients with DOC (Schiff et al., 2007; Giacino et al., 2012; Della Pepa et al., 2013; Yamamoto et al., 2013; Cossu, 2014; Thibaut et al., 2014; Tucker and Sandhu, 2016).

Transcranial direct current stimulation (tDCS) is a promising non-invasive brain stimulation technique for treatment of patients with DOC, which is safe, less uncomfortable and easy to handle (Zhang and Song, 2018). tDCS modulates cortical excitability at stimulation sites via weak current which flows through the brain from the anode to the cathode. Anodal tDCS boost neuronal activation via sub-threshold neuronal membrane polarization, and cathodal tDCS can reduce cortical excitability (Lefaucheur et al., 2017). The effects of tDCS stimulation can be long-lasting, and are connected with the duration of stimulation and current intensity (Zhao et al., 2017). Previous studies illustrated the residual capacity for neural plasticity and recovery of consciousness in some patients with DOC. Our group showed that tDCS can effectively modulate the cortical excitability of patients with DOC, especially in patients with MCS (Bai et al., 2017a,c). Martens et al. found that 4 weeks of home-based tDCS moderately improved the recovery of signs of consciousness in patients with MCS (Martens et al., 2018). Similarly, Zhang and his colleagues reported 20 sessions of tDCS can improve CRS-R scores and modulate the P300 amplitude in patients with MCS. The P300 has been commonly used to detect residual awareness in patients with DOC (Giacino et al., 2018). Several studies have also showed that patients with MCS can benefit from tDCS over the left dorsolateral prefrontal cortex (DLPFC) (Angelakis et al., 2014; Thibaut et al., 2014, 2015, 2017; Dimitri et al., 2017). Except the treatment effect of tDCS in patients with DOC, tDCS induced changes in cortical connectivity and excitability is useful in differentiating MCS from UWS patients (Naro et al., 2015). However, another study discovered that tDCS of the left DLPFC did not have remarkable clinical and EEG effects in patients with DOC (Estraneo et al., 2017). A critical problem of tDCS in patients with DOC is stimulated area. The left DLPFC is common in traumatic brain injury, Huang et al. (2017) chose the posterior parietal cortex as the site of stimulation. Researchers found tDCS of the posterior parietal cortex improves the recovery of clinical signs of consciousness in some patients with MCS (Huang et al., 2017). For researching the mechanism of change of tDCS in patients with DOC, a more focal stimulation is important. However, The main disadvantage of conventional tDCS is that it produces diffuse brain current flow. It is difficult to interpret whether produced effects are due to stimulation of the targeted cortical region or neighboring anatomical area (Bai et al., 2014; To et al., 2016).

High-Definition tDCS using the 4 × 1 smaller compact scalp electrodes, instead of the two large pad electrodes, is a new neuromodulation technique. HD-tDCS improves the spatial precision, resulting in focal neural and specific behavioral changes (Dmochowski et al., 2011; Villamar et al., 2013; Shekhawat and Vanneste, 2018). HD-tDCS has been previously reported to improve motor function, verbal learning, working memory, and pain and tinnitus control (Borckardt et al., 2012; Caparelli-Daquer et al., 2012; Kuo et al., 2013; Donnell et al., 2015; Nikolin et al., 2015; Shekhawat et al., 2016). It has been shown to reliably target specific brain areas and produce plastic changes that may outlast conventional tDCS (Kuo et al., 2013; Hogeveen et al., 2016). There is no study has examined the impact of HD-tDCS on DOC to date.

The CRS-R is a standardized behavioral assessment measure that has been widely used for diagnostic assessment and outcome measurement in patients with DOC (Gerrard et al., 2014). However, the rate of clinical misdiagnosis based on the CRS-R remains high (Xie et al., 2017). Previous results have demonstrated EEG can detect and analyze brain activity in clinical practice (Lehembre et al., 2012; Bai et al., 2017a,b). Our previous research has found the quantitative EEG was useful for assessment of the effect of tDCS and rTMS in patients with DOC (Bai et al., 2017a,c; Xia et al., 2017). EEG coherence has been applied to evaluate the effective connectivity of DOC, a high coherence hints at an increased functional interplay between the underlying neuronal networks (Rampil, 1998; Davey et al., 2000; Bai et al., 2017a,c).

We applied resting state EEG and CRS-R scores for assessing the effect in patients with DOC treated with long-lasting HD-tDCS. We aim to confirm that HD-tDCS applied to the precuneus on patients with DOC could produce clinically useful behavioral modifications. We also want to find direct EEG evidence to demonstrate the efficacy of HD-tDCS in patients with DOC.

## Materials and Methods

### Patients

We enrolled medically stable 18 patients with DOC hospitalized in Department of Neurosurgery, Zhengzhou Central Hospital Affiliated to Zhengzhou University, between October 2016 and June 2017. Due to pulmonary infection, phlebothrombosis, and other clinical interferences, 11 patients (5 VS and 6 MCS, mean age:52.8 years, range: 30.0–71.0 years, 4 females, and 7 males) completed the entire experiment (Table 1). Inclusion criteria were VS/UWS or MCS patients, according to the JFK CRS-R scores. We excluded patients with DOC who had precuneus lesions, have had tDCS treatment before or last less than 3 months to avoid the spontaneous recovery period. Participants who had pacemakers, aneurysm clips, other devices implanted or other treatments and drugs which modifying cortical-excitability were also eliminated. The present study was approved by the ethics committee of the Zhengzhou Central Hospital Affiliated to Zhengzhou University.

**Table 1:** Demographic details of the patients included in the study.

				Duration
Patient	Age	Etiology	MRI findings	(months)	CRS-R						
					A	V	M	OM	C	Ar	Total
MCR1	66–70	Hemorrhage	Left frontal-parietal lesions, diffuse atrophy	3	2	3	3	1	0	2	11
MCR2	51–55	Hemorrhage	Left frontal-temporal lesion	3	2	2	2	1	0	1	8
MCR3	56–60	Hemorrhage	Left hemisphere lesion, diffuse cortical atrophy	3	2	3	2	1	0	2	10
MCR4	26–30	Hemorrhage	Left parietal-temporal-thalamus lesion	6	1	2	2	1	0	1	7
MCR5	71–75	Hemorrhage	Left frontal-temporal-parietal lesions	4	2	2	3	1	0	2	10
MCR6	51–55	Hemorrhage	Right frontal-parietal-thalamus lesions	3	1	2	2	1	0	2	8
VS1	36–40	Hemorrhage	Left frontal -temporal cortical atrophy	6	1	1	2	0	0	2	6
VS2	61–65	Hemorrhage	Left hemisphere lesion	3	1	1	2	1	0	1	6
VS3	51–55	Hemorrhage	Right frontal-temporal cortical atrophy	3	1	1	2	1	0	1	6
VS4	36–40	TBI	Bilateral frontal and diffuse cortical atrophy.	8	1	1	2	1	0	1	6
VS5	51–55	TBI	Left frontal-temporal, diffuse cortical atrophy	6	1	0	2	0	0	1	4

*CRS-R, Coma Recovery Scale–Revised scores; A, auditory; V, visual; M, motor; OM, oromotor; C, communication; Ar, arousal.*

### Design and HD-tDCS: Stimulation Protocol

**FIGURE 1:**
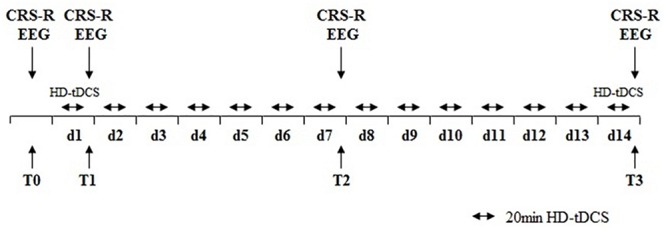
Protocol of the study. HD-tDCS, high-definition transcranial direct current stimulation; CRS-R, Coma Recovery Scale–Revised scores; EEG, electroencephalography.

All patients received HD-tDCS modulation (2 mA, 20 min, anode centered over the precuneus) for two session per day over 14 consecutive days (Figure 1). The CRS-R assessments were conducted at four time points: before the experiment (T0), after a single session of HD-tDCS (T1), after the treatment of 7 days (T2), and 14 days (T3). In this study, any side effects of HD-tDCS were monitored and reported.

### HD-tDCS

4 x1-Ring high-definition electrodes with an anode center electrode overlying the targeted brain area surrounded by four cathodal electrodes were used to deliver direct current to the scalp with the application of Ag/AgCl ring electrodes (Model 4x1-C2: Soterix Medical Inc., New York, NY, United States). HD-tDCS enables a more restricted cortical neuromodulation and leads to higher electric fields. Electrodes were held in place by specially designed plastic casings embedded in a 32-channel EEG recording cap. The center electrode (anode) was placed at Pz according to the international 10–20 EEG system, and four cathodal electrodes were placed approximately 3.5 cm radially from Pz; corresponding roughly to locations Cz, P3, P4, and POz (Figure 3A).

### EEG Recording and Pre-processing

We used 32 EEG recorder (Nicolet EEG V32, Natus, United States). EEG recorded at four time points: before the experiment (T0), after a single session of HD-tDCS (T1), after the treatment of 7 days (T2), and 14 days (T3). EEG signals were continuously recorded from 32 channels at positions of the International 10/20 system. The electrodes with the setting of a band-pass filtered at DC to 1000 Hz in the recorder. The EEG signal was digitized at a sampling rate of 2.5 kHz. The skin impedance was maintained below 5 kΩ. EEG recordings were carried out while patients were behaviorally awake.

Off-line analysis was carried out using EEGLAB 12.0.2.5b, running in a MATLAB environment (version 2013b, Math Works Inc., Natick, Massachusetts, United States). The 50-Hz power signal was removed by a notch filter. The independent component analysis function was used to identify and remove the artifact-relevant components. The EEG data were down-sampled to 500 Hz and average referenced. Then, the EEG date were divided into epochs of 10 s with 50% overlap in each patient.

### EEG Analysis

Coherence was measured using spectral cross-correlation and normalized power spectra of signals obtained from two electrodes with the following equation:

$$\begin{array}{c} {\text{Coh}}_{\text{xy}}\left(f\right)=\frac{{\left|P_{\text{xy}}\left(f\right)\right|}^{2}}{P_{\text{xx}}\left(f\right)P_{\text{yy}}\left(f\right)} \end{array}$$

where P_xy_(f) was the cross-power spectral density and P_xx_(f) and P_yy_(f) were the respective auto-power spectral densities of the signals.

**FIGURE 2:**
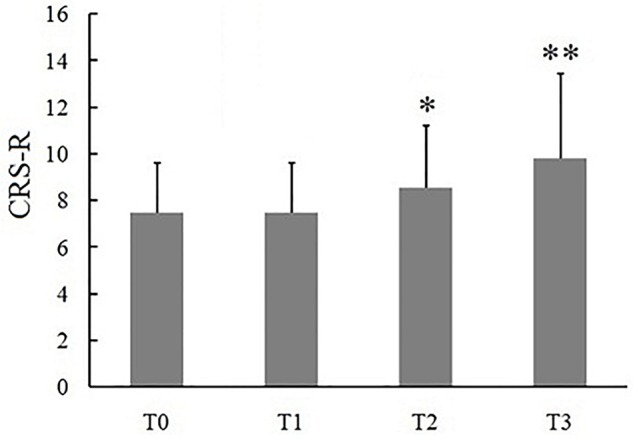
Effects of long-lasting HD-tDCS treatments evaluated with CRS-R in patients with DOC. Asterisk indicates significant differences (^∗^P < 0.05, ^∗∗^*P* < 0.01).

As shown in Figure 2A, the frontal region included electrodes Fp1, Fp2, Fz, F3, F4, Fc5, Fc6, Fc1, FC2, F7, and F8; the central region included electrodes CZ, C3,C4,Cp1,Cp2,Cp5, and Cp6; the parietal region included electrodes Pz, P3, P4, and Poz. Central-parietal coherence was calculated using pairwise electrodes from the central and parietal regions. The frontal inter-hemisphere and central inter-hemisphere coherences were also calculated.

### Statistics

The statistics were performed via SPSS for Windows, version 17.0. The Wicoxon signed-rank test was used to analyze the effects of HD-tDCS on CRS-R. And the Kolmogorov–Smirnov test was utilized to observe the coherence between different regions at the delta bands. Bonferroni correction was conducted after multiple comparisons. P < 0.05 was regarded as statistically significant.

## Results

### Effects of the HD-tDCS Treatment as Measured by CRS-R

Eleven (6 MCS and 5 VS) patients with chronic DOC completed the treatment, with no specific side effects, such as redness of the skin, signs of discomfort or epilepsy. 9/11 (72%) patients (54% of responders, 6 MCS and 3 VS) showed the CRS-R scores increased after 14 days of stimulation (Table 2). The CRS-R scores increased with the treatment going on, compared with the baselines, and the CRS-R score at the 7 day was significantly higher than the baseline (Figure 2, P < 0.05). It demonstrated that long-lasting HD-tDCS treatment can improve the recovery of consciousness in patients with DOC, whereas behavioral changes were not observed at just one session of stimulation.

**Table 2:** Clinical evaluation of the patients on day 7 and 14.

	CRS-R improvement						
Patient	(day 7/14)							Changes of diagnosis
	A	V	M	OM	C	Ar	Total	
MCR1	0/1	0/0	0/0	0/0	0/0	0/0	0/1	MCS- elevated to MCS+
MCR2	0/0	1/1	1/1	0/0	0/0	1/1	3/3	Remained MCS-
MCR3	0/1	0/0	1/2	0/1	0/1	0/0	1/5	MCS- elevated to MCS+
MCR4	0/2	1/1	0/1	0/0	0/1	1/1	2/6	MCS- elevated to MCS+
MCR5	0/0	0/1	0/0	0/0	0/0	0/0	0/1	Remained MCS-
MCR6	1/2	1/1	1/1	1/2	0/0	0/0	4/6	MCS- elevated to MCS+
VS1	0/0	0/0	0/0	0/0	0/0	0/0	0/0	Remained VS
VS2	0/0	1/1	0/0	0/0	0/0	0/1	1/2	Remained VS
VS3	0/0	0/1	0/0	0/0	0/0	0/0	0/1	Remained VS
VS4	0/0	0/0	0/0	0/0	0/0	1/1	1/1	Remained VS
VS5	0/0	0/0	0/0	0/0	0/0	0/0	0/0	Remained VS

*CRS-R, Coma Recovery Scale–Revised scores; A, auditory; V, visual; M, motor; OM, oromotor; C, communication; Ar, arousal.*

### Effects of the HD-tDCS Treatment as Measured by EEG

**FIGURE 3:**
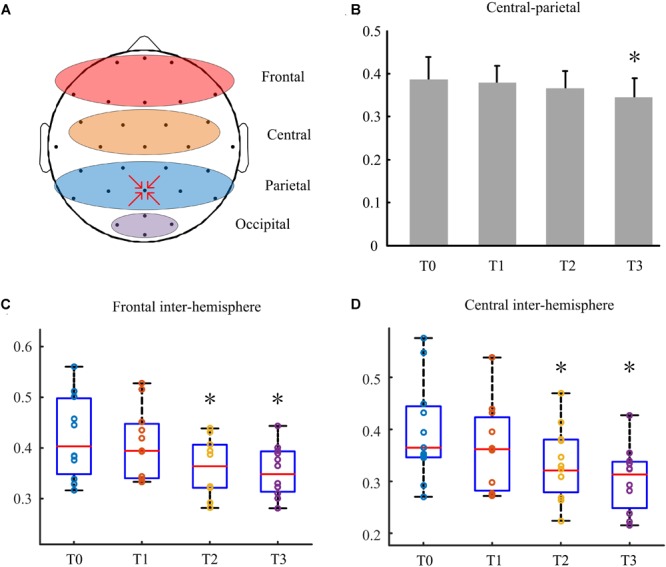
The coherence between different regions in the delta bands. **(A)** Defined different regions of brain. **(B)** The coherence between central and parietal regions. **(C)** The frontal inter-hemisphere coherence. **(D)** The central inter-hemisphere coherence. Asterisks indicate statistically significant differences (^∗^P < 0.05).

The coherence in the delta bands between the defined central and parietal regions was calculated (Figure 3B). Results showed that it decreased with the treatment going on, and the coherence index at T3 was higher significantly than that at T0 (P < 0.05). Figure 3C showed behavioral improvement was not discovered after the first stimulation. But the delta band coherence changed in the frontal inter -hemisphere regions in some patients with DOC. Besides, it reduced remarkably at day 7 (P < 0.05). Similarly, as shown in Figure 3D, the coherence between the central inter- hemisphere regions reduced after stimulation, and especially on day 7 and 14 (P < 0.05).

## Discussion

Several studies have reported effectiveness of conventional tDCS over the left DLPFC in patients with DOC (Angelakis et al., 2014; Thibaut et al., 2014, 2015, 2017; Dimitri et al., 2017). Patients with MCS but not VS are more easily benefit from tDCS at the left DLPFC. Recently, a sham-controlled randomized clinical trial investigated that 9/37 (27%) patients with MCS showed improvements after conventional tDCS over the posterior parietal cortex (Huang et al., 2017). A randomized double-blinded sham-controlled cross-over study didn’t support effectiveness of conventional tDCS over the left DLPFC in patients with DOC (Estraneo et al., 2017). Estraneo et al.’s (2017) study didn’t observe relevant behavioral and EEG changes in the single or repeated stimulation over the left DLPFC. What’s more, owing to low spatial resolution of conventional tDCS, it was difficult to explain causality between stimulation of target brain region and the behavioral changes (Kuo et al., 2013). In addition, many studies only used CRS-R to evaluate treatment effects of conventional tDCS. In fact behavioral changes are not always observed in patients with DOC, particularly in short-term modulation. The precuneus as known to be involved in conscious processes plays an important role in consciousness recovery (Laureys and Schiff, 2012). Therefore, we targeted anodal HD-tDCS at the precuneus to evaluated modulation clinical effects and EEG oscillation in patients with DOC. We found that long-lasting HD-tDCS improved the recovery of consciousness in patients with MCS and some patients in VS. Resting state EEG showed a significant reduction of the coherence between the central and parietal region at the delta band. Significant decreasing of coherence was also found at inter-hemisphere of frontal and central. These changes occurred in different time windows and brain regions for patients with DOC. No side effects such as discomfort, skin burn, and seizures were observed after any of the stimulation.

High-Definition tDCS delivery system was developed to enhance the spatial accuracy of tDCS, which is believed to enhance the clinical effects of this therapeutic tool. HD-tDCS uses the 4 × 1 montage of stimulating electrodes, which generates in maximal focused electric field strength under the target electrode with brain current flow constrained by the ring radius (Kuo et al., 2013; Gbadeyan et al., 2016; Hogeveen et al., 2016). Thus, it produced more spatially restricted electric field, as compared to the conventional electrode placement. HD-tDCS has the characteristics of high spatial resolution and more focused electric field than conventional tDCS protocols, which may offer the opportunity to explore the contribution of stimulation cortical target to consciousness. The efficacy of HD-tDCS for investigating motor cortex excitability, conscious movement intention, fibromyalgia, pain, tinnitus, verbal learning, and memory functioning have been reported (Borckardt et al., 2012; Caparelli-Daquer et al., 2012; Kuo et al., 2013; Donnell et al., 2015; Nikolin et al., 2015; Shekhawat et al., 2016). To our knowledge, there is no study has examined the impact of HD-tDCS on DOC. Targeting the precuneus using HD-tDCS will help probably to understand the recovery mechanisms of clinical sign of consciousness better.

The site of stimulation is also a critical scientific issue (Xia et al., 2017). The left DLPFC, cerebellum and the posterior parietal cortex were selected as the stimulation sites in DOC frequently. Cerebellum involves in short- and long-term habituation of unconditioned responses (Naro et al., 2016; Bocci et al., 2018), but it is not essential for consciousness. Cerebellar tDCS may be useful for ameliorating the level of consciousness (Naro et al., 2016). Both Left DLPFC and the precuneus are involved in conscious processes (Schiff, 2010; Xia et al., 2017). Conventional tDCS of the DLPFC have shown promising results in patients with MCS, which requires gray matter integrity (Thibaut et al., 2015). The probability of damage in DLPFC is higher than the posterior parietal cortex in DOC, the latter seems a better stimulation site in clinic (Pandya and Seltzer, 1982). The precuneus is associated with memory retrieval, controlling spatial aspects of behavior and Visual-spatial visualization (Wenderoth et al., 2005; Ionta et al., 2014; Blanke et al., 2015; Kragel and Polyn, 2016; Rissman et al., 2016). The precuneus plays an important role in the mesocircuit model. Thibaut et al. (2015) found the precuneus metabolism and behavioral level supporting the fronto-parietal network correlate with outcomes in DOC (Thibaut et al., 2015). Moreover, the precuneus seems to be a brain region that can differentiate patients with MCS from VS.

As mentioned in the introduction, a single session of tDCS over the left DLPF transiently improves CRS-R total scores in patients with MCS (Giacino et al., 2012). Results of another study suggest that repeated tDCS improves the recovery of consciousness in 56% patients with DOC (Thibaut et al., 2017). In this study, We didn’t observe any patient showed behavioral response to HD-tDCS after the first session of stimulation. Interestingly, we observed that 6/11 patients (54% of responders,4 MCS and 2 VS) showed the CRS-R scores significant improvement after 7 days of stimulation. The improvement was observed in 6 patients with MCS and 3 patients with VS after 14 days of stimulation. Four patients with MCS – rose from MCS - to MCS +. These results suggested that repeated HD-tDCS daily could promote consciousness level in MCS, whereas all VS remained previous consciousness state. These results suggested that the first session is not predictive of a future positive effect of the stimulation on the level of consciousness. Stimulation term is another critical issue, long-lasting stimulation possible improves neuroplasticity and strengthen the effect of the stimulation. In the future, longer-term stimulations (such as 20,30, or 60 days) should be considered to discover the potential of recovery effects in patients with DOC. MCS patients have more prominent potential of neural plasticity, which attain more benefit from HD-tDCS.

To reveal the mechanism of action and clinical effects of HD-tDCS over the precuneus of patients with DOC, functional connectivity of coherence was investigated using based resting state EEG. Previous studies have shown that the severity of DOC was correlated with increased low-frequency band power and decreased high-frequency band power (Bai et al., 2017a,b,c; Xia et al., 2017). Our data showed the significant reduction of short-range central-parietal coherence at the delta band after long-lasting HD-tDCS modulation. The long-range frontal-parietal coherence in the delta band did not decrease. But the frontal inter-hemisphere coherence significantly decreased in the delta band with increasing stimulation sessions. Besides, remarkably decrease compared to baseline was first shown at day 7. Similar results were observed in the central inter-hemisphere. Behavioral improvement was not discovered after the first stimulation. But the delta band coherence changes in brain implied a cortical response to the stimulation. Some patients who had no response to the first stimulation CRS-R scores improved after the whole stimulation session. These indicated that HD-tDCS could effectively alter the brain electrical activity. Accordingly, HD-tDCS induced variation of delta band coherence negatively correlated with the patients’ CRS-R scores to some extent.

Studies demonstrated that the degree of DOC may be correlated with increased low-frequency band power in EEG patients (Bai et al., 2017a,b,c; Xia et al., 2017). The alteration of delta oscillations is accompanied by function alterations in the brain (Cavinato et al., 2015). The rationale for performing HD-tDCS in consciousness recovery remains unclear, but we found the trend of changes in the delta band in the frontal central and parietal regions. These changes can be summarized in a modulation of cortical coherence in short-range central-parietal and long-range frontal-parietal areas within a delta frequency range. Therefore, we considered that the changes occurring in the delta bands may provide evidence for supporting the modulating effects of HD-tDCS in patients with DOC.

Our study has several limitations. Firstly, the sample size was small. In the following research, we need recruit more patients with DOC to confirm and validate tDCS effectiveness. Secondly, this study lacked a randomized cross-over design and follow-up assessment, for long-term effect needs to be verified to determine its clinical effect. Thirdly, initial level of consciousness varied from VS/UWS to MCS. Therefore, future clinical trials should set up MCS and VS groups based on larger samples. In addition, we did not utilize neurophysiological and neuroimaging technology (e.g., event-related potential ERP, mainly the P300 component, functional MR), which would allow to better understand the treatment effects and mechanisms of HD-tDCS in patients with DOC (Zhang et al., 2017; Ragazzoni et al., 2019). What’s more, P300 recording reflecting residual levels of awareness can assist in prognostication regarding 12-month recovery of consciousness for patients with DOC (Giacino et al., 2018). In future study, Multi-Modality technology should be applied together to provide a broader and more holistic evaluation of therapeutic efficacy.

## Conclusion

In this study, we found that Long-lasting HD-tDCS over the precuneus could improve the recovery of consciousness in patients with DOC. EEG changes in the delta band were observed in fronta-central-parietal cortex, which provides direct evidence of the HD-tDCS protocol effects on the patients with DOC. Further studies are needed to verify the clinical effect of HD-tDCS on larger numbers of patients and expound the mechanism of the recovery of consciousness of HD-tDCS protocol.

## Data Availability

All datasets generated for this study are included in the manuscript and/or the supplementary files.

## Ethics Statement

This study was carried out in accordance with the recommendations of the ethics committee of the Zhengzhou Central Hospital with written informed consent from all subjects. All subjects gave written informed consent in accordance with the Declaration of Helsinki. The protocol was approved by the ethics committee of the Zhengzhou Central Hospital.

## Author Contributions

YWD, JH, CL, and HZ designed the study. YG, JL, and XW collected the data. YB, XX, and YYD analyzed the data. YB and CL created the figures and tables. YG wrote and edited the manuscript.

## Conflict of Interest Statement

The authors declare that the research was conducted in the absence of any commercial or financial relationships that could be construed as a potential conflict of interest.
